# Cost-effectiveness of three malaria treatment strategies in rural Tigray, Ethiopia where both Plasmodium falciparum and Plasmodium vivax co-dominate

**DOI:** 10.1186/1478-7547-9-2

**Published:** 2011-02-08

**Authors:** Hailemariam Lemma, Miguel San Sebastian, Curt Löfgren, GebreAb Barnabas

**Affiliations:** 1 Tigray Health Bureau, P.O. box 7, Mekelle, Ethiopia; 2 Umeå International School of Public Health, Dept of Public Health and Clinical Medicine, Umeå University, 901 85 Umeå, Sweden

## Abstract

**Background:**

Malaria transmission in Ethiopia is unstable and the disease is a major public health problem. Both, *p.falciparum *(60%) and *p.vivax *(40%) co-dominantly exist. The national guideline recommends three different diagnosis and treatment strategies at health post level: i) the use of a *p.falciparum*/*vivax *specific RDT as diagnosis tool and to treat with artemether-lumefantrine (AL), chloroquine (CQ) or referral if the patient was diagnosed with *p.falciparum*, *p.vivax *or no malaria, respectively (parascreen pan/pf based strategy); ii) the use of a *p.falciparum *specific RDT and AL for *p.falciparum *cases and CQ for the rest (paracheck pf based strategy); and iii) the use of AL for all cases diagnosed presumptively as malaria (presumptive based strategy). This study aimed to assess the cost-effectiveness of the recommended three diagnosis and treatment strategies in the Tigray region of Ethiopia.

**Methods:**

The study was conducted under a routine health service delivery following the national malaria diagnosis and treatment guideline. Every suspected malaria case, who presented to a health extension worker either at a village or health post, was included. Costing, from the provider's perspective, only included diagnosis and antimalarial drugs. Effectiveness was measured by the number of correctly treated cases (CTC) and average and incremental cost-effectiveness calculated. One-way and two-way sensitivity analyses were conducted for selected parameters.

**Results:**

In total 2,422 subjects and 35 health posts were enrolled in the study. The average cost-effectiveness ratio showed that the parascreen pan/pf based strategy was more cost-effective (US$1.69/CTC) than both the paracheck pf (US$4.66/CTC) and the presumptive (US$11.08/CTC) based strategies. The incremental cost for the parascreen pan/pf based strategy was US$0.59/CTC to manage 65% more cases. The sensitivity analysis also confirmed parascreen pan/pf based strategy as the most cost-effective.

**Conclusion:**

This study showed that the parascreen pan/pf based strategy should be the preferred option to be used at health post level in rural Tigray. This finding is relevant nationwide as the entire country's malaria epidemiology is similar to the study area.

## Background

Malaria continues to be a global challenge with half of the world's population at risk of the disease. In 2006 about 250 million episodes of malaria occurred globally with nearly a million deaths, mostly of children under 5 years of age. More than 85% of this disease burden was concentrated in countries in Sub-Saharan Africa (SSA). Ethiopia was one of the five main contributors to the overall African malaria burden [1,2].

In Ethiopia, despite the long history of malaria control since the 1950s, the disease is still a major public health problem[3]. Though some improvements, both in mortality and morbidity, have been recently achieved, malaria has been consistently reported as one of the three leading causes of morbidity and mortality over the past years [4]. Malaria in Ethiopia is seasonal, predominantly unstable and focal, depending largely on rainfall and altitude. Two transmission seasons are known: major (September to December) and minor (April to May). The unstable nature of malaria makes the population non-immune and prone to focal and cyclical epidemics. Unlike most SSA countries where *p.falciparum *almost accounts for all malaria infection, in Ethiopia, both *p.falciparum and p.vivax *are co-dominant, the former accounting for approximately 60% of all cases. In the low transmission season *p.vivax *increases its proportion due to its relapsing nature and the seasonal drop in *p.falciparum *infection [3,5,6].

In fighting against this deadly disease, early diagnosis and prompt treatment is one of the most basic and effective global strategies [7,8]. The effectiveness of this strategy is highly dependent on the national policy of providing effective diagnosis and first-line antimalarial drugs, and in the delivery system.

In 2004, Ethiopia made two important policy changes which favoured this strategy. Firstly, it launched a community-based health care system, the Health Extension Programme (HEP), to achieve significant essential health care coverage. HEP is the grass-root level of the primary health care (PHC) through the provision of two health extension workers (HEWs) in a health post (HP) at tabia (sub-district) level to serve approximately 5,000 inhabitants. HEWs are high school graduated women with one year of training on the components of the HEP programmes. HEP is a package of sixteen basic health components. All components of the programme comprise health promotion and prevention activities except the malaria intervention which, in addition, incorporates diagnosis and treatment [9]. HEP has been successfully implemented throughout the country including Tigray. Currently, there are more than 1,220 health extension workers in Tigray and the coverage has increased from 30% in 2006/7 to above 70% in 2007/8 [10].

Secondly, the country has made two changes on its national malaria diagnosis and treatment guideline. Malaria confirmatory diagnosis using rapid diagnostic tests (RDTs) replaced presumptive diagnosis, while maintaining the latter approach where the former is unavailable [8]. A presumptive malaria case is a patient who exhibits fever or history of fever within the past 48 hrs in the absence of clear symptoms indicating alternative causes of fever. RDTs are tests based on the detection of antigens released from the malaria parasites in lysed blood [11]. The second change included a shift in the treatment of *p.falciparum *from monotherapy sulphadoxine-pyrimethamine (SP) to artemisinin-based combination therapy (ACT), namely artemether-lumefantrine (AL), while keeping chloroquine (CQ) for treating *p.vivax*. The guideline recommends three different diagnosis and treatment strategies: i) if malaria is diagnosed with a falciparum-specific and pan-specific device, treat *p.falciparum *cases with AL, *p.vivax *with CQ and refer negatives to a higher level; ii) if malaria is diagnosed with only a *p.falciparum*-specific device, treat positive (*p.falciparum*) cases with AL and all the remaining with CQ; and iii) if malaria is diagnosed presumptively, treat all suspected cases with AL [8]. *P.falciparum *positive patients for whom AL is contra-indicated have to be treated with quinine and patients with one or more signs and symptoms of severity should be referred immediately to the nearest higher facility.

In the study year, 2007, on top of the presumptive diagnosis, two types of RDTs were in use at the health-post level in the study area: parascreen pan/pf (Zephyr Biomedical, Goa, India) and paracheck pf (Orchid Biomedical Systems, Goa, India); the former is able to identify both *p.falciparum *and *p.vivax *while the latter targets only *p.falciparum*. While paracheck pf was the commonly used RDT at health post level since 2004, parascreen pan/pf had been recently introduced.

Several studies on RDT cost-effectiveness (CE) have been conducted in the past years. Most of these studies were focused in areas of high malaria transmission and *p.falciparum*. Almost all were comparing potentially similar types of RDTs either with microscope and/or presumptive diagnosis [12-18]. However, none of them are similar to the Ethiopia malaria epidemiological context and to the current national diagnosis and treatment strategies.

Therefore, this operational research was designed to assess the cost-effectiveness of the recommended three diagnosis and treatment strategies in the Tigray region of Ethiopia. This will provide evidence to assist decision makers on which strategy is the most appropriate in the region

## Methods

### Study area

Tigray regional state is located in northern Ethiopia and is divided into 47 woredas (districts). The region has approximately 4.3 million inhabitants most of whom (81.2%) live in rural areas [19]. The majority of the population works in agriculture. Famine and drought are regular occurrences in the region. As in the rest of Ethiopia, malaria transmission in Tigray is very seasonal and occurs mainly at altitudes up to 2,200 meters above sea level (masl). Around 65% of the population is at risk of malaria and the disease was the number one cause of outpatient cases, admissions and deaths. In 2006, it accounted for 28% of all the patients treated in the regions' health facilities [20]. Previous efforts to control the problem have included insecticide residual spraying and environmental management. Since 2005, distribution of long-lasting insecticidal nets is gradually covering all malarious villages.

The health system in Tigray is essentially the same as in the rest of Ethiopia, i.e., a four-tier system with Primary Health Care Units (PHCUs) at the grass-roots level. There are five zone-level hospitals, six district hospitals and one referral hospital in Mekelle, the capital.

### Sampling procedure

In order to capture epidemiological variations, the study was stratified into the three commonly known malaria strata in the country: stratum-I (<1000 masl), stratum-II (1000-1500 masl) and stratum-III (1501-2000 masl). A district in a given stratum with a high number of villages was selected to represent its respective stratum. Four districts were selected: Kafta-Humera, Tahtay-Adiyabo, and Mereb-leke plus Raya-Azebo from strata I, II, and III respectively. Two districts were included in the strata III for being the largest strata. The districts populations ranged from 91,379 in Tahtay-Adiyabo to 136,039 in Raya-Azebo. In all the study districts, malaria has constituted a leading cause of the disease burden over years. For instance, in 2007/8 it accounted for 21%-28% of outpatient visits in the districts [20].

The study was conducted under a routine HEP service following the national malaria diagnosis and treatment guideline during the main transmission months of 2007. Half of the health posts (7-8) in each district were randomly selected.

#### Patient enrolment and management

All diagnosis and treatment procedures were done by the HEWs under routine conditions following the national guideline. HEWs (enumerators) were trained with a major focus on how to interpret the result of the newly introduced parascreen pan/pf device, blood film preparation and data collection. No additional training was given on paracheck pf as it had been used for years.

Every suspected malaria case, who presented to a HEW either at a village or health post, was included. Following the national malaria guideline, patients were excluded if they: i) exhibited signs and symptoms of severe malaria or any other severe disease, ii) had taken antimalarial drugs (AL or quinine) within the previous two weeks, and iii) were infants under three-months-old or were pregnant mothers during their first trimester for whom AL is contraindicated.

Previous years have shown a slide positivity rate (SPR) of approximately 30% in the high-transmission season [20]. For this anticipated SPR, with a confidence level of 95%, an absolute precision of five percentage points (25% to 35%) and a design effect of two), the required sample size was 646 patients for each stratum.

Patient history, including demographic data, signs and symptoms related to current illness (chief complaint) and medication, was collected. A finger-pricked blood sample from each subject was taken for the two types of RDTs, according to the RDT manufacturer's instructions (leaflet enclosed within the kit) and a blood film (thick and thin) for the microscope examination following the World Health Organization (WHO) guideline [21]. Patients were treated for malaria if one of the RDTs was positive.

### The reference expert microscopy

Performance of the three alternative diagnostic and treatment strategies were calculated vis-à-vis the light microscopy. Blood films were stained with 3% Giemsa stain and examined by two independent (first and second) microscopists using ×1000 oil immersion following the WHO guideline [21]. The independent readings were compared for concordance of presence or absence of asexual/sexual forms of *plasmodium *and its species. A third senior microscopist examined discordant slides and his/her findings taken as true diagnostic outcome. A negative was declared after 200 microscopic fields read without finding a parasite. The first and the second microscopists were unaware of the RDT results and the third reader was blind to the results of both the RDTs and the preceding microscopists.

### Data analysis

The cost-effectiveness (CE) of the three different diagnosis and treatment strategies was compared. The strategies included: i) the use of parascreen pan/pf as diagnosis tool and to treat with AL, CQ or referral if the patient was diagnosed with p.falciparum, p.vivax or no malaria respectively (parascreen pan/pf based strategy); ii) the use of paracheck pf and AL for p.falciparum cases and CQ for the rest (paracheck pf based strategy); and iii) the use of AL for all cases diagnosed presumptively as malaria (presumptive based strategy). All data were entered in to Microsoft Excel version 8. Effectiveness was calculated using Epi Info™ version 3.5 [22] and the cost and cost-effectiveness were calculated using Microsoft Excel 8.

#### Costing

Costing was undertaken from the provider's perspective (government) at the health post level and restricted only to the first visit of a patient. At this facility level, the entire malaria diagnosis and treatment service is free of charge.

Costing considered only diagnosis and antimalarial drugs because the fixed costs (infrastructure, supervision, training and HEWs salaries) were assumed not to differ among the comparative strategies. The cost of these items is also shared with other health programmes. RDT provision, compared to presumptive diagnosis, comprises other operational and management costs at different levels in addition to the cost of the test kit; however, this cost was reasonably assumed as similar for both RDT-based strategies and traded-off with the expenditure reduction on drug management and transport as RDT application decreased the amount of AL needed. RDT costing was at the manufacturer's price and was calculated as the total number of presumptive patients multiplied by the unit price of each type of RDT kit (including lancets, swabs, pipette, buffer solution and desiccant).

AL costing was calculated at the manufacturer's cost (but not CQ) as it has been provided at no profit, as per the special pricing agreement between WHO and the manufacturer [23]. Antimalarial drug cost was calculated following the malaria diagnosis and treatment guideline at the peripheral level. Being age dependent, the number of cases in each treatment regimen was multiplied by the cost of the respective treatment course of either AL or CQ. Unit costs were obtained from the Tigray Health Bureau (THB) pharmacy unit for the year 2007. The following items were not including in costing: RDT reading time, RDT wastage and RDT training cost.

#### Effectiveness indicator and cost-effectiveness measure

RDTs, highly specific and less sensitive compared to presumptive diagnosis, are mainly introduced since presumptive treatment is non-specific while it is 100% sensitive. Therefore, there is a need to balance the risk between improving specificity (excluding non-malaria cases) and reducing sensitivity (missing malaria cases) while replacing presumptive with RDTs. Taking this into account, we selected the number of correctly treated cases (CTC) as the measure of effectiveness on the basis of the malaria diagnosis and treatment strategies. This indicator accommodates both concerns: detecting the malaria cases (sensitivity) and excluding the non-malaria cases (specificity) supporting the public health goal of properly managing all causes of illness. In low malaria prevalence areas such as Tigray [24], all malaria infections, even with low-level parasitaemia, are associated with clinical illness in all age groups. In such malaria epidemiology, there is no evidence if missing malaria cases is more or less dangerous than missing non-malaria cases or the vice-versa. Therefore, it was assumed that the weight of correctly or mistakenly treating cases of any disease including malaria was equal. A non-malaria case identified by the parascreen pan/pf was referred to a higher health facility level - this meant that this patient was correctly treated. The number of correctly treated cases was then calculated as the number of true positives plus the number of true negatives cases.

Cost-effectiveness was estimated as average cost-effectiveness ratio (ACER) and incremental cost-effectiveness ratio (ICER). ACER was calculated as a cost of diagnosis and treatment of a given strategy divided by the number of CTCs. To find out if an extra cost in a strategy produced an extra effect (health benefit), ICER analysis was conducted where the strategies were ranked by increasing cost and then the additional cost in one strategy was divided by the additional CTCs [25].

#### Sensitivity analysis

Sensitivity analysis for selected parameters for which the cost-effectiveness is more sensitive was conducted. Changes in some variables may have skewed some findings; to allow for this, a one-way sensitivity analysis was carried out on changes in AL cost and SPR. A reduction in cost for AL was incorporated into the analysis since the price of AL has been constantly decreasing throughout the last few years (even though drug resistance may necessitate the purchase of more expensive antimalarial drugs in the future). We did not consider changes on RDT price, as it seems unlikely to drop in the near future for at least two possible reasons: firstly, there is a huge gap between the demand and supply - for instance, in 2006, only 16 million RDTs were distributed while 80 million courses of ACTs were used [1]. Secondly, despite the potential high demand, the prices have been kept constant in the last years.

Change in SPR as a function of seasonal variation is inevitable. We considered a minor transmission season (the point estimate was of the major season), whilst assuming the diagnostic performance remained constant. A two-way sensitivity analysis was also carried out at a reduced AL cost during a low transmission season.

### Ethical clearance

Ethical clearance was obtained from Tigray Health Bureau, Mekelle, Ethiopia. District Health Offices were informed of the study and its purposes. The purpose of the study was explained to the participants. Verbal consent was obtained from (patient/patient's guardian) as the majority of the rural population is illiterate. No patient refused to participate. Confidentiality of patient identity was maintained for every enrolled patient by assigning a unique identification number that was labelled on the RDT devices, blood film slides, data collection forms and database.

## Results

### Characteristics of the subjects

In total 2,422 subjects from all three strata and 35 health posts were enrolled in the study. Overall, 26.63% (n = 645), 28.0% (n = 677) and 45.42% (n = 1100) of the subjects were from strata I, II and III, respectively. In total, 37.2% (n = 901) were female, 13.96% (n = 338) were children aged under five years, 18.66% (n = 452) aged between 5-14 years and the remaining 67.38% were 15 years or above. The age of the study subjects ranged from three months to 85 years with a mean of 24.18 (median of 21 years). Eighteen percent of them sought treatment within one day since the onset of illness. Most of the patients (86.21%) appeared with fever and the remaining with a history of fever.

### Microscope result

The microscope examination of thick blood smear showed a crude (all species and all stages) SPR of 27.29% (n = 661) with 68.53% (n = 453) of the positive samples being *p.falciparum (+/-*gametocytes, gametocyte alone and mixed) and 31.47% (n = 208) *p.vivax (+/- gametocytes*) (Table 1). The stratified SPR was 46.51%, 26.88% and 16.27% with a *p. falciparum *proportion of 68%, 69.78% and 69.77% for stratum I, II and III, respectively. There were 27 cases of gametocytes, out of which 26 were in the presence of asexual stage. There was only one mixed infection of *p.falciparum *and *p.vivax*. From the operational point of view, all these 28 cases were considered as *p.falciparum*. There was one case of *p.vivax *in the presence of gametocytes which was considered as *p.vivax*.

**Table 1:** Summary result of the comparison between the expert microscopy and the RDTs, Tigray, Ethiopia, 2007.

Expert Microscope	Paracheck pf		Parascreen pan/pf		Total (microscope)
	**Positive**	**Negative**	**Positive**	**Negative**	

P.falciparum					
Positive	402	51	377	76	453
Negative	114	1855	97	1872	1969
Total	516	1906	474	1948	2422
P.vivax					
Positive	-	-	155	53	208
Negative	-	-	53	2161	2214
Total	-	-	208	2214	2422

P.falciparum positive is: asexual +/- sexual, asexual +/- p.vivax; P.vivax positive is: asexual +/- sexual; Paracheck pf negative is meant no-p.falciparum; Parascreen pan/pf p.vivax positive meant non p.falciparum malaria

### Cost analysis

The unit cost was US$ 0.59 (US$ = 9.00 Ethiopian birr for 2007) for the paracheck pf kit, US$1.05 for the parascreen pan/pf kit and US$ 0.03 for a pair of gloves. A treatment course of AL cost US$ 0.60, 1.20, 1.80 and 2.40 according to the treatment regimen (age) group. Each CQ tablet cost US$0.006.

The cost analysis indicated that the presumptive-based strategy (BS) was higher by 27.69% and 46.1% than the cost of the parascreen-BS and paracheck-BS, respectively. In the RDT-BS, the tests' cost accounted for the majority of the expenditure, 55.52% in paracheck-BS and 72.08% in parascreen-BS. AL constituted 41%, 27.65% and 100% of the total cost of paracheck-BS, parascreen-BS and presumptive-BS, respectively. Cost of chloroquine was insignificant which was 3.48% in paracheck-BS and less than 1% in parascreen-BS.

### Effectiveness indicator and cost-effectiveness

Out of the 661 malaria and 1761 non-malaria cases, parascreen-BS correctly treated 88.48% cases (377 *p.falciparum*, 155 *p.vivax *and 1611 negatives) (Table 2). It failed to identify 11.52% patients, out of which 5.33% were malaria patients (76 *p.falciparum *and 53 *p.vivax*) who would have been left untreated (false negatives) and 6.19% (97 false *p.falciparum *and 53 false *p.vivax*) would have been incorrectly given antimalarial drugs (Table 1). Paracheck-BS correctly treated 23.95% cases (402 *p.falciparum *and 178 *p.vivax*) and mislabelled 76.05% (n = 1842). Out of these, 3.34% were malaria (51 *p.falciparum *classified as *p.vivax *and 30 *p.vivax *as *p.falciparum*) and 72.70% (n = 1761) were non-malaria (114 cases classified as *p.falciparum *out of which 11 were *p.vivax *and 1647 as *p.vivax *when they were not). The presumptive-BS captured all the *p.falciparum*, (18.7%, n = 453) but mistreated 1969 cases (81.30%) as *p.falciparum*, out of which 8.59% (208) were *p.vivax *and 72.71% were non-malaria (Table 2).

**Table 2:** Effectiveness and cost ($US) of the three different diagnostic strategies, Tigray, Ethiopia, 2007

Description	Different treatment strategies		
	**Presumptive ** **n (%)**	**Paracheck-BS ** **n (%)**	**Parascreen-BS ** **n (%)**

Correctly treated *p.falciparum *cases	453	402	377
Correctly treated p*.vivax *cases	0	178	155
Correctly treated non-malaria cases	0	0	1611
Total correctly treated cases	453 (18.70)	580 (23.95)	2143 (88.48)
Test Cost	0	1501.64(55.52)	2615.76(72.08)
AL cost	5017.2	1108.80(41.00)	1003.20(27.65)
CQ cost	0	94.05(3.48)	9.80(0.27)
Total cost	5017.20	2704.49	3628.76

The CE analysis showed that the parascreen-BS was the most cost-effective with ACER US$ 1.69/CTC followed by US$ 4.66/CTC for the paracheck-BS and US$11.08/CTC for the presumptive-BS (Table 3). ICER analysis was conducted to find out whether this additional cost was worth paying to get the added effect. Presumptive-BS was highly dominated (less effect for more money) by parascreen-BS. Therefore, the ICER calculation was limited to parascreen-BS over paracheck-BS. At the base case, the additional cost on parascreen-BS over paracheck-BS would be able to treat an additional 64.5% (n = 1563) of patients correctly with an incremental cost of US$0.59/patient.

**Table 3:** Average and incremental cost-effectiveness ratios among the three diagnosis strategies, Tigray, Ethiopia, 2007.

Diagnostic based strategy	Cost	Correctly treated cases	ACER	Incremental cost	Incremental effect	ICER	Remark
Paracheck	2704.5	580	4.66	-	-	-	
Parascreen	3628.8	2143	1.69	924.27	1563	0.59	
Presumptive	5017.2	453	11.08	1388.44	-1690	-0.82	dominated

### Sensitivity analysis

Taking into account the AL cost in the International Drug Price Indicator (2008 version) that showed a reduction of 32.8% (lowest dose), 33.25% (for the middle doses) and to 36.9% (adult dose) [26], a sensitivity analysis revealed a high reduction in the cost of the presumptive-BS by 37.14%, in paracheck-BS by 14.93% and in parascreen-BS by 10.05% (Table 4). The base case ACER was improved by 36.20% (from US$11.08 $US7.05) in presumptive-BS, by 14.81% (from US$ 4.66 to $US 3.97) in paracheck-BS and by 10.05% (from US$ 1.69 to $US 1.52) in parascreen-BS. Despite the significant drop in ACER, presumptive-BS was still dominated by parascreen-BS. The ICER of parascreen-BS over paracheck-BS was increased from $US0.59 to $US0.62 for each additional 1563 correctly treated cases.

**Table 4:** Sensitivity analysis at reduced AL cost and low-transmission for three malaria diagnostic strategies, Tigray, 2007

	At reduced AL price			Low transmission season (15% SPR)		
**Comparison**	**Strategy**			**Strategies**		

	**Paracheck-BS**	**Parascreen-BS**	**Presumptive-BS**	**Paracheck-BS**	**Parascreen-BS**	**Presumptive-BS**

Base case cost	2704.49	3629.76	5017.20	2704.49	3629.76	5017.2
Total new cost	2300.80	3264.20	3192.70	1926.05	2908.56	5017.20
Cost change within strategy	403.69	364.56	1886.50	778.44	720.20	0
Cost change in (%)	(14.93)	(10.05)	(37.14)	(28.78)	(19.85)	0
Correctly treated cases (n, %)	580 (23.95)	2143 (88.48)	453 (18.7)	315 (13.01)	2253 (93.02)	127 (5.24)
ACER at reduced AL and SPR	3.97	1.52	7.05	6.11	1.29	39.51
ACER Change from base case, %	(14.81)	(10.06)	(36.2)	(+31.1)	(23.67)	(+258)
Cost difference b/n strategy	0.00	963.40	-71.50	0.00	982.51	2108.64
Effect difference b/n strategy	0.00	1563.00	-1690	0.00	1938	-2124
ICER	-	0.62	dominated	-	0.51	dominated

+ ACER indicates higher value than the base case

The sensitivity analysis at 15% SPR during the minor transmission season with 35% *p.falciparum *to 65% *p.vivax*, with no change in the diagnostic performance of the strategies to the base case, showed a reduction in the proportion of correctly treated cases in the presumptive and paracheck-BS. The proportion of CTC was, however, increased in the parascreen-BS strategy (Table 4). The base case ACER decreased in parascreen-BS (from $US 1.69 to $US 1.29/CTC) and increased in the paracheck (from $US 4.66 to $US 6.11/CTC) and presumptive-BS (from $US 11.08 to $US 39.51/CTC) per correctly treated case. This illustrated that the cost-effectiveness increased by 23.67% in the parascreen-BS, decreased in the paracheck-BS by 31.12% and deteriorated significantly in the presumptive-BS by 258%. Since presumptive-BS was dominated, the IECR was recalculated as parascreen-BS over paracheck-BS. The base case of $US 0.59 dropped to $US 0.51/additional correctly treated case (Table 4).

A two-way sensitivity analysis (Table 5) at reduced cost of AL during the minor transmission season showed an increase in the ACER from $US 4.66 to $US 5.75 and from $US 11.08 to $US 25.14 in the paracheck-BS and presumptive-BS, respectively, while it dropped from $US 1.69 to $US 1.25 in the parascreen-BS. The two-way sensitivity analysis showed that presumptive-BS continued to be dominated by parascreen-BS.

**Table 5:** A two-way sensitivity cost-effectiveness analysis at reduced cost of AL during low-transmission season, Tigray, 2007.

Diagnostic strategies	Cost	Correctly treated cases	ACER	Incremental cost	Incremental effect	ICER
Paracheck-BS	1812.61	315	5.75	0	0	0
Parascreen-BS	2806.27	2253	1.25	993.66	1938	0.51
Presumptive-BS	3193.00	127	25.14	386.73	-2126	-0.18 (dominated)

## Discussion

This is, to our knowledge, the first empirical study in Ethiopia evaluating the economic implications of the malaria diagnostic and treatment strategies currently implemented in the country. It is also a unique study in that it compared two RDTs targeting different plasmodium-specific antigens (*p.falciparum *and *p.vivax *vs. only *p.falciparum*) from an operational point of view.

This study has supported two central facts regarding the malaria transmission pattern in the region: firstly, our result of SPR (27.3%) and species composition of *p.falciparum *to *p.vivax *(68.5% to 31.5%) is highly consistent with the commonly quoted statistics in serial reports of the THB [20]. Secondly, it has confirmed that malaria in the region varies from place to place due to differing altitude. The SPR was 46.51% for the lower stratum, 26.88% for the middle, and 16% for the highest stratum whilst showing a similar proportion of *p.falciparum *to *p.vivax *(range 68%-69.78%). As many other studies have indicated [17,27,28], this research has also revealed that the shift from presumptive-BS to RDT-BS is clearly of significant benefit in the era of ACT. In our context where malaria transmission is low, the likelihood of a fever episode being due to malaria, even during the peak transmission season, is on average 30%. Approximately one-third of this corresponds to *p.vivax*, increasing to two-thirds during the minor transmission season. The prevalence and proportion of the species bitterly challenges the presumptive-BS strategy as it leads to mistreat numerous *p.vivax *and false non-malaria cases. The need of using RDT-BS is therefore not debatable. Instead, the discussion should be tailored toward which type of RDT is the more cost-effective to ensure the maximum number of patients receive appropriate treatment. Accordingly, parascreen-BS was found to be the more cost-effective. The ICER showed that, if we invest in parascreen-BS instead of paracheck-BS, we can properly manage 65% (1563) additional cases for as little as $ 0.59/patient. If we spend on presumptive-BS instead of parascreen-BS, the cost rises to US$ 0.82/patient (highly dominated). In fact, the cost-effectiveness of RDT-BS over presumptive-BS was partially increased at the expense of some missed malaria cases, since the RDTs are less sensitive than the presumptive-BS. We are also aware that if the effectiveness measure would have been only malaria cases, the paracheck-BS would have been the more cost-effective. However, the health benefit with the parascreen-BS is higher as more non-malaria cases get appropriate treatment and the saving is greater by avoiding over prescription. Over-treatment of malaria results in considerable morbidity and mortality by delaying the correct treatment of non-malaria illness and by contributing to the development and spread of antimalarial resistance strains.

The sensitivity analysis showed that the cost-effectiveness of the strategies varied depending on the season and AL cost. With the AL price drop, all alternatives improved their cost-effectiveness; however, in the low-transmission season, both the paracheck-BS and the presumptive-BS suffered while the parascreen-BS still improved. This shows that parascreen-BS is even more cost effective with reduced AL cost and during low-transmission season, which is the longest period of the year (December-August). Though no sensitivity analysis was made with regard to the different malaria strata, the higher the elevation, the lower the SPR makes parascreen pan/pf still more cost effective. Studies conducted in semi-immune populations have shown a higher cost effectiveness of RDTs in children <5 years compared to other age groups [14,29]. In our case, where all age groups share practically equal risk of malaria, this sensitivity analysis was not relevant.

Though the literature on the cost-effectiveness of RDTs has been growing in the last years [12-18,28], no comparable study designs to ours were found. The focus of all the studies has been on one type of plasmodium-specific RDT, either *p.falciparum *specific [13,14,17] or in combination with *p.vivax *[12,16]. Our study compared both types of plasmodium-specific RDTs at the same time.

## Methodological considerations

There are some considerations to take into account which can potentially affect the findings of this research. Firstly, our study was limited to the health-provider perspective at the rural health post level. If a full societal perspective had been used to capture the distributional impact of the intervention, the epidemiological and economical advantages of the best RDT-BS might have been even higher. One limitation was that the study design did not allow us to capture whether HEWs complied with the guideline in their therapeutic decision-making. The HEW prescription report might not show the actual practice. Experience from the field and recent studies have shown that health workers are prescribing antimalarial drugs regardless of negative test results [17,30-35].

Cost calculation did not include the RDT reading time, RDT wastage and RDT training cost. The former is difficult to measure because the reading time might include attending several patients. RDTs could be wasted for different reasons such as poor transport, storage conditions and due to inappropriate use. To estimate these wastage's costs would have been extremely difficult. The few hours training on RDT, which it is a long-term investment, made also difficult to allocate the cost to the patients. In our study, weights to malaria and non-malaria cases were assumed to be equal since our study population is non-immune. In some studies conducted in semi-immune populations, more weight has been given to non-malaria patients because malaria severity is less in adults [13,28].

## Conclusions

The study has shown that the most cost-effective strategy was the one which used parascreen pan/pf in the treatment of malaria. The finding is relevant not only for Tigray region but also for the whole country, since malaria epidemiology follows a similar pattern nationally. Since 2008, the only available strategies at the health post level in the country have been the paracheck pf-BS and presumptive-BS. Our finding, pointing the superiority of the parascreen pan/pf based strategy, call decision-makers to reconsider this policy.

These results will be, however, pertinent only if an adequate supply of RDT and first-line antimalarial drugs at the health-post level are ensured and if HEWs comply with test results. Furthermore, and importantly, proper management of RDTs and adequate training and continuous supervision of HEWs should also be maintained. Finally, a study that captures the final health outcome of malaria diagnosis and treatment strategies and assesses HEWs' compliance with test results should be top research priorities in the region

## Conflict of interests

The authors declare that they have no competing interests.

## Authors' contributions

HL developed the study design, collected and analysed data and drafted the manuscript. MSS, CL, GB contributed to the study design and critically read and improved the manuscript. All authors read and approved the final manuscript.

## References

[B1] WHO World Malaria Report 2008 2008 Geneva: The World Health Organization 215

[B2] RBM/WHO Global Malaria Action Plan for a malaria-free world 2009 Geneva: Roll Back Malaria Partnership 45

[B3] FMoH National five-years strategic plan for malaria prevention & control in Ethiopia, 2006-2010 2006 Addis Ababa: Federal Democratic Republic of Ethiopia, Minstry of Health

[B4] Otten M Aregawi M Were W Karema C Medin A Bekele W Jima D Gausi K Komatsu R Korenromp E Low-Beer D Grabowsky M Initial evidence of reduction of malaria cases and deaths in Rwanda and Ethiopia due to rapid scale-up of malaria prevention and treatment Malar J 2009 8 14 10.1186/1475-2875-8-14 19144183 PMC2653503

[B5] Deressa W Ali A Enqusellassie F Self-treatment of malaria in rural communities, Butajira, southern Ethiopia Bull World Health Organ 2003 81 4 261 268 12764492 PMC2572446

[B6] Ghebreyesus TA Witten KH Getachew A Yohannes AM Tesfay W Minass M Bosman A Teklehaimanot A The community-based malaria control programme in Tigray, northern Ethiopia. A review of programme set-up, activities, outcomes and impact Parassitologia 2000 42 3-4 255 290 11686085

[B7] WHO Antimalarial drug combination therapy 2001 Geneva: The World Health Organization

[B8] FMoH Malaria diagnosis and treatment guideline for health workers in Ethiopia, 2nd edition 2004 Addis Ababa: Federal Democratic Republic of Ethiopia, Ministry of Health

[B9] Health Extension Programme-Major training packages http://cnhde.ei.columbia.edu/training/index.html

[B10] San Sebastian M Lemma H Efficiency of the health extension programme in Tigray, Ethiopia: a data envelopment analysis BMC Int Health Hum Rights 2010 10 16 10.1186/1472-698X-10-16 20546597 PMC2893104

[B11] WHO New Perspectives, Malaria Diagnosis 2000 Geneva: The World Health Organization 57

[B12] de Oliveira MR de Castro Gomes A Toscano CM Cost effectiveness of OptiMal(R) rapid diagnostic test for malaria in remote areas of the Amazon Region, Brazil Malar J 2010 9 277 10.1186/1475-2875-9-277 20937094 PMC2959076

[B13] Rolland E Checchi F Pinoges L Balkan S Guthmann JP Guerin PJ Operational response to malaria epidemics: are rapid diagnostic tests cost-effective? Trop Med Int Health 2006 11 4 398 408 10.1111/j.1365-3156.2006.01580.x 16553923

[B14] Lubell Y Reyburn H Mbakilwa H Mwangi R Chonya K Whitty CJ Mills A The cost-effectiveness of parasitologic diagnosis for malaria-suspected patients in an era of combination therapy Am J Trop Med Hyg 2007 77 6 Suppl 128 132 18165484

[B15] Wiseman V Kim M Mutabingwa TK CJM W Cost-effectiveness study of three antimalarial drug combinations in Tanzania PLoS Medicine 2006 3 10 10.1371/journal.pmed.0030373 PMC1592341 17032059

[B16] Bualombai P Prajakwong S Aussawatheerakul N Congpoung K Sudathip S Thimasarn K Sirichaisinthop J Indaratna K Kidson C M S Determining cost-effectivness and cost component of three malaria diagnostic modules being used in remote non-micrscope areas South Asian J Trop Med Public Health 2003 34 2 322 33 12971557

[B17] Chanda P Castillo-Riquelme M Masiye F Cost-effectiveness analysis of the available strategies for diagnosing malaria in outpatient clinics in Zambia Cost Eff Resour Alloc 2009 7 5 10.1186/1478-7547-7-5 19356225 PMC2676244

[B18] Lubell Y Hopkins H Whitty CJ Staedke SG Mills A An interactive model for the assessment of the economic costs and benefits of different rapid diagnostic tests for malaria Malar J 2008 7 21 10.1186/1475-2875-7-21 18226224 PMC2266929

[B19] CSA The 2007 Population and housing census results of Ethiopia 2007 Addis Ababa: Central Statistical Agency of Ethiopia

[B20] THB Tigray Health profile 2004-2008 2004 Mekelle: Tigray Health Bureau

[B21] WHO Basic Malaria Microscopy. Part I Learner's guide 1991 Geneva: The World Health Organization

[B22] CDC Epi Info™ version 3.5.1 2008 Epi Info™ version 3.5.1. Atlanta, USA: Centers for Disease Control and Prevention

[B23] Procurement of artemether/lumefantrine (Coartem^®^) through WHO http://archives.who.int/tbs/access/CoA_website5.pdf

[B24] WHO The community-based malaria control programme in Tigray, Ethiopia. A review of programme set-up, activities, outcomes, and impact. Tigray. Report WHO/CDS/RBM/99.12 1999 Geneva: THB/WHO/Roll Back Malaria 11686085

[B25] Drummond ME O'Brien B Stoddart GL Torrance GW Methods for Economic Evalution of Health Care Programmes 1997 2 Oxford: Oxford University Press

[B26] International drug price indicator http://erc.msh.org/dmpguide/index.cfm?searc

[B27] Uzochukwu BS Obikeze EN Onwujekwe OE Onoka CA Griffiths UK Cost-effectiveness analysis of rapid diagnostic test, microscopy and syndromic approach in the diagnosis of malaria in Nigeria: implications for scaling-up deployment of ACT Malar J 2009 8 265 10.1186/1475-2875-8-265 19930666 PMC2787522

[B28] Shillcutt S Morel C Goodman C Coleman P Bell D Whitty CJM Mills A Cost-effectiveness of malaria diagnostic methods in sub-Saharan Africa in an era of combination therapy Bul World Health Organ 2008 86 2 101 110 10.2471/BLT.07.042259 PMC2647374 18297164

[B29] Zikusooka CM McIntyre D Barnes K Should countries implementing an artemisinin-based combination malaria treatment policy also introduce rapid diagnostic tests? Malar J 2008 7 176 10.1186/1475-2875-7-176 18793410 PMC2556342

[B30] Bisoffi Z Gobbi F Angheben A Van den Ende J The role of rapid diagnostic tests in managing malaria PLoS Med 2009 6 4 e1000063 10.1371/journal.pmed.1000063 19399160 PMC2667642

[B31] Reyburn H Mbakilwa H Mwangi R Mwerinde M Olomi R Drakeley C Whitty CJM Rapid diagnostic tests compared with malaria microscopy for guiding outpatient treatment of febrile illness in Tanzania: randomised trial BMJ 2007 334 403 406A 10.1136/bmj.39073.496829.AE 17259188 PMC1804187

[B32] Hamer DH Ndhlovu M Zurovac D Fox M Yeboah-Antwi K Chanda P Sipilinyambe N Simon JL Snow RW Improved diagnostic testing and malaria treatment practices in Zambia JAMA 2007 297 20 2227 2231 10.1001/jama.297.20.2227 17519412 PMC2674546

[B33] Bisoffi Z Sirima BS Angheben A Lodesani C Gobbi F Tinto H Van den Ende J Rapid malaria diagnostic tests vs. clinical management of malaria in rural Burkina Faso: safety and effect on clinical decisions. A randomized trial Trop Med Int Health 2009 14 5 491 498 10.1111/j.1365-3156.2009.02246.x 19222821

[B34] Lubell Y Reyburn H Mbakilwa H Mwangi R Chonya S Whitty CJ Mills A The impact of response to the results of diagnostic tests for malaria: cost-benefit analysis BMJ 2008 336 202 205 10.1136/bmj.39395.696065.47 18199700 PMC2213875

[B35] Msellem MI Martensson A Rotllant G Bhattarai A Stromberg J Kahigwa E Garcia M Petzold M Olumese P Ali A Björkman A Influence of rapid malaria diagnostic tests on treatment and health outcome in fever patients, Zanzibar: a crossover validation study PLoS Med 2009 6 4 10.1371/journal.pmed.1000070 PMC2667629 19399156

